# Correlation of Alzheimer’s Disease Death Rates with Historical Per Capita Personal Income in the USA

**DOI:** 10.1371/journal.pone.0126139

**Published:** 2015-05-11

**Authors:** Dariusz Stępkowski, Grażyna Woźniak, Marcin Studnicki

**Affiliations:** 1 Laboratory of Molecular Basis of Cell Motility, Nencki Institute of Experimental Biology, Warszawa, Poland; 2 Department of Experimental Design and Bioinformatics, Warsaw University of Life Sciences-SGGW, Warszawa, Poland; Texas Tech University Health Science Centers, UNITED STATES

## Abstract

Alzheimer’s disease (AD) is a progressive degenerating disease of complex etiology. A variety of risk factors contribute to the chance of developing AD. Lifestyle factors, such as physical, mental and social activity, education, and diet all affect the susceptibility to developing AD. These factors are in turn related to the level of personal income. Lower income usually coincides with lower level of education, lesser mental, leisure—social and physical activity, and poorer diet. In the present paper, we have analyzed the correlation of historical (1929–2011) per capita personal income (PCPI) for all states of the USA with corresponding age-adjusted AD death rates (AADR) for years 2000, 2005 and 2008. We found negative correlations in all cases, the highest one (R ≈ -0.65) for the PCPIs in the year 1970 correlated against the AADRs in 2005. From 1929 to 2005 the R value varies in an oscillatory manner, with the strongest correlations in 1929, 1970, 1990 and the weakest in 1950, 1980, 1998. Further analysis indicated that this oscillatory behavior of R is not artificially related to the economic factors but rather to delayed biological consequences associated with personal income. We conclude that the influence of the income level on the AD mortality in 2005 was the highest in the early years of life of the AD victims. Overall, the income had a significant, lifelong, albeit constantly decreasing, influence on the risk of developing AD. We postulate that the susceptibility of a population to late-onset AD (LOAD) is determined to a large extent by the history of income-related modifiable lifestyle risk factors. Among these risk factors, inappropriate diet has a significant contribution.

## Introduction

The prevalence of Alzheimer’s disease (AD) is steadily increasing worldwide, including the USA [1,2,3]. The reasons for such a profound increase in the number of AD cases are unclear. In order to elucidate the causes of this increase, risk factors present in the history of a population should be considered. Among known factors increasing the risk of developing Alzheimer’s disease the most important one is the aging of the population [4,5]. Alzheimer’s disease is therefore most probably related to the aging processes on the cellular and organismal level. Other pathologies prevalent in the population that increase the risk of dementia including AD are diabetes [6,7], hypertension [8], high level of cholesterol and other factors related to vascular health [8,9], strokes [10], midlife obesity [11], bad diet [12,13,14,15], depression [16], head injuries [17], and exposure to toxins [18]. Smoking [19], lower socioeconomic status [20,21], physical inactivity [22,23,24], low level of education of patients and their parents [20,25,26], mental inactivity [22,24] and lack of social engagement [27] also contribute to the risk of developing dementia and AD. The strength of evidence and the relevance to the susceptibility to AD of some of these risk factors is discussed in [28]. Some authors point to early-life events as the most important ones for shaping the future susceptibility of an individual to AD [29]. Some of the risk factors listed are interrelated and this makes historical analysis of their influence on the population extremely difficult. However, many of them can be grouped together as lifestyle factors significantly related to the economic status. It is possible to track per capita personal income (PCPI) data for the population of the United States of America, from before the Great Depression of the 1930s when the majority of present Alzheimer’s patients were born. To check if, and how, the PCPI, presumably by affecting the lifestyle, has an influence on the AD mortality, we studied the correlation of PCPI for each state of the USA, starting from the year 1929, just before the Great Depression, through the year 2011, with age-adjusted AD death rates (AADRs) for those states in 2000, 2005 and 2008.. We found a significant negative correlation of PCPI with AADRs for all the years studied. Despite the presence of many potential confounders such as migration between states, historical lifestyle differences between the states, differences in climate, rural to urban population ratios, different stratification of personal income etc., PCPI seems to be a convenient parameter discriminating between healthier and less healthy lifestyles.

## Methods

The PCPI data for each state for years 1929–2011 were taken from the FRED St. Louis data base (http://research.stlouisfed.org/series) and compiled into an Excel worksheet (available in S1 File). For each year, linear regression was performed with AADRs for years: 2000, 2005 and 2008 using Origin 7.5 software (AADRs were taken from National Vital Statistics Reports, Deaths final data for 1998–2010). For all three sets of data on AADR, the highest correlation was obtained with PCPI for the year 1970. Regressions were done using PCPI for each state normalized to PCPI in 2011 using the following formula: PCPI in a given year * Fn where Fn = PCPI (2011) (average US in 2011) /PCPI in a given year (average US in that year). This normalization allows for comparison of the slope for each year, without affecting the correlation coefficient R—an indicator of the strength of correlation. This normalization is different from the recalculation of the income in constant dollars, which gives real values of the income but does not permit to compare slopes of correlations from different years. The absolute value of the slope of the correlation between PCPI and AADR can be interpreted as the number of dollars by which a decrease in PCPI causes one additional AD-linked death per 100 000 population. The higher the absolute value of the slope the steeper the relation of death rates with PCPI. It means that an increase in the death rate by one victim of AD per 100 000 population is related to a higher decrease in PCPI. The normalization used by us allows the slopes of the correlations from different years to be compared with each other despite the differences of the real value of dollars corresponding to those slopes. Using constant dollars recalculated to the value of 2011 dollars would not allow such a comparison due to differences in the level of income. The average US PCPI in constant dollars for each year in the period 1929–2011 was calculated using the US inflation calculator (http://www.usinflationcalculator.com/) based on the Consumer Price Index for urban consumers prepared by the U.S. Department of Labor, Bureau of Labor Statistic. Pearson’s coefficient R was chosen as the measure of the strength of correlation over R^2^ since it differentiates between negative and positive correlation and offers higher sensitivity (due to the wider range of variability (-1,1) than that of R^2^ (0,1)). Additionally, unlike R^2^, R changes linearly with the change in data values therefore, R is more suitable for use to relate the variability of R with the susceptibility of population to AD.

### Statistical methods

A crucial question about the statistical significance of the oscillatory nature of R variability and the trend lines (for the correlations between PCPI and age-adjusted AD death rates (AADRs) in 2000, 2005,2008) was addressed by dividing the studied period into seven segments (seasons). These segments correspond to periods between the peaks of consecutive highest and lowest correlations (extremes). The first segment spans the period between 1929 and 1936, the second from 1937 to 1950, the third from 1951 to 1969, the fourth from 1970 to 1980, the fifth from 1981 to 1991, the sixth from 1992 to 2002 and the seventh the period from 2003 to 2011. For each period the Mann-Kendall test and value of tau correlation were calculated. These tests were used to statistically assess the monotonicity of upward or downward trends of R variability in time. The results of these tests are presented in Supporting Information 2. Furthermore, the 95% confidence interval from polynomial regressions (degree 16) was used to determine the statistical significance of the differences between the extreme (minima and maxima) values of the regression curve. The polynomial regressions for AADR in 2000, 2005 and 2008 were performed using the R 3.1.2 software with lm function. The 95% confidence intervals are presented as the upper and lower confidence borders for the designated polynomial functions and were based on Student's t distribution. The pattern of division of the total studied period and the validity of extremes of the regression curve were confirmed by differential analysis. Differential analysis was performed by subtraction of the R value for a given year from the R value of the preceding year. These differentials were plotted against time and regressed by polynomial regression procedure similarly as for correlations between PCPI and AADR. The first derivative function obtained in this way zeroed in most cases of the extreme values of the original curve, confirming both the validity of segmental division and of the extreme points of the original correlations (minima and maxima).

### Rationale

The most important risk factor for developing AD is aging [4,5]. The percentage of people 85 and over with AD and other dementias in the USA is the highest among all susceptible age groups and is reported to be 30.2 for whites, 58.6 for African-Americans and 62.9 for Hispanics [5]. This leads to the conclusion that the aging process is a significant contributors to the etiology of this disease, whereas socioeconomic or lifestyle-related biological factors and genetic susceptibility factors differentiating races may have a modifying effect [30]. It is well known that diet affects the risk of developing AD [12,13,14,15]. Diet, by its nature, is highly income-dependent within a given cultural setting. Therefore, we used per capita personal income (PCPI) as a possible indicator of the likelihood of a healthy lifestyle, assuming that higher income increases the likelihood of more healthy behaviors, including a more healthy, diversified diet. We chose the data of AD death rates, available for each state of the USA, as more amenable to analysis than the prevalence or incidence data, which are based on estimations from small populations and may be biased due to improper sampling. Throughout the period chosen, 1998–2008, the USA ADDRs data are comparable owing to the application of the same system of issuing death certificates (ICD10) (National Vital Statistics Reports, Deaths final data for 1998–2010). We used age-adjusted data to compensate for the different age structure in the populations of the US states. These data exclude deaths from other primary causes but with co-existing AD (a discussion of this problem can be found in a recent report of the Alzheimer’s Association [5]). We assumed that the proportion between AD deaths as primary cause and deaths from other diseases with coexisting AD was constant in the studied period. Therefore, underrepresentation of AD in death certificates does not influence conclusions from our studies. One possible bias of our analysis can be caused by the increased life span during the period studied (from 76.7 in 1998 to 77.4 in 2005—National Vital Statistics Reports. Deaths final data for 1998–2010) which could increase the proportion of deaths due to AD as the primary cause in relation to other deaths. However, since the life expectancy change was only minor, the effect of the increase in the life span on the AADRs should also be negligible. Other possible sources of bias and their relation to the quality of our analysis are presented in the Discussion.

## Results

### Negative correlation of PCPI with age-adjusted death rates

**Fig 1:**
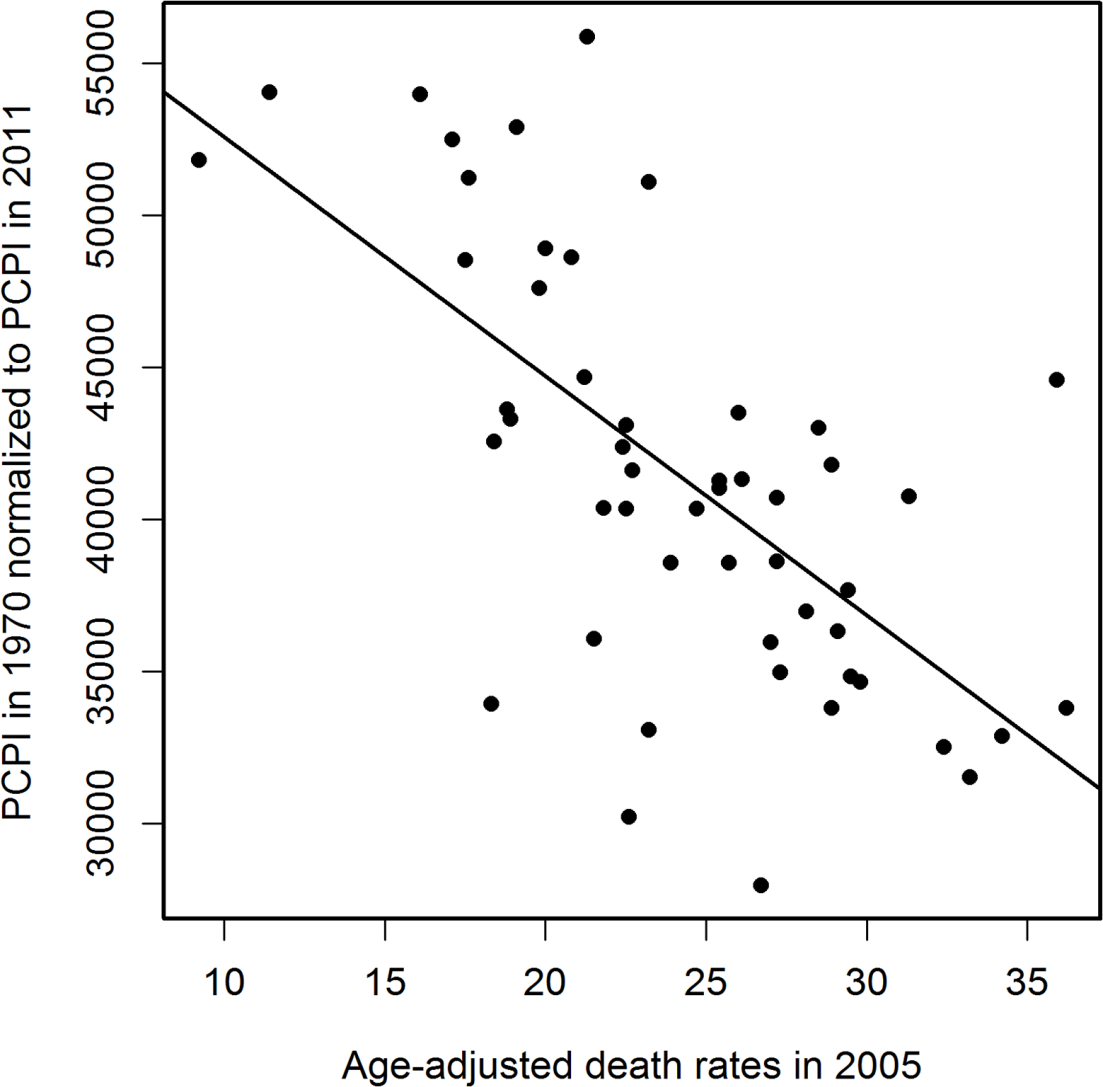
Negative correlation of PCPI in 1970 (in 51 states of the USA) with age-adjusted AD death rates (AADRs) for the respective states in 2005. Death rates are represented by the number of cases per 100 000 population adjusted for age distribution.

**Fig 2:**
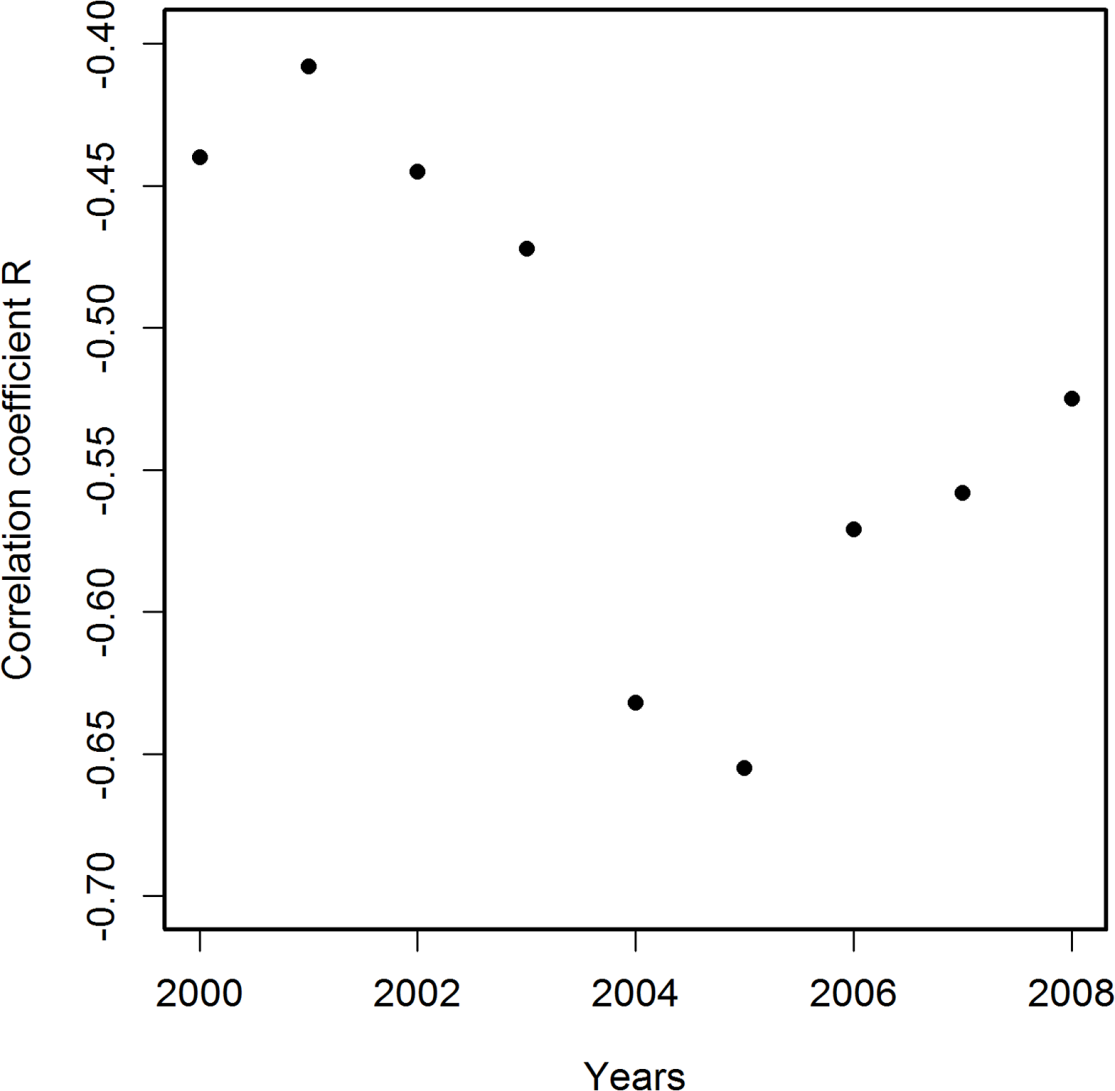
Correlation coefficients R for correlations of PCPI in 1970 (in 51 states of the USA) with age-adjusted AD death rates (AADRs) in the respective states in the period 2000–2008. The best correlation is observed for deaths data for 2005. Death rates are represented by the number of cases per 100 000 population adjusted for age distribution.

**Fig 3:**
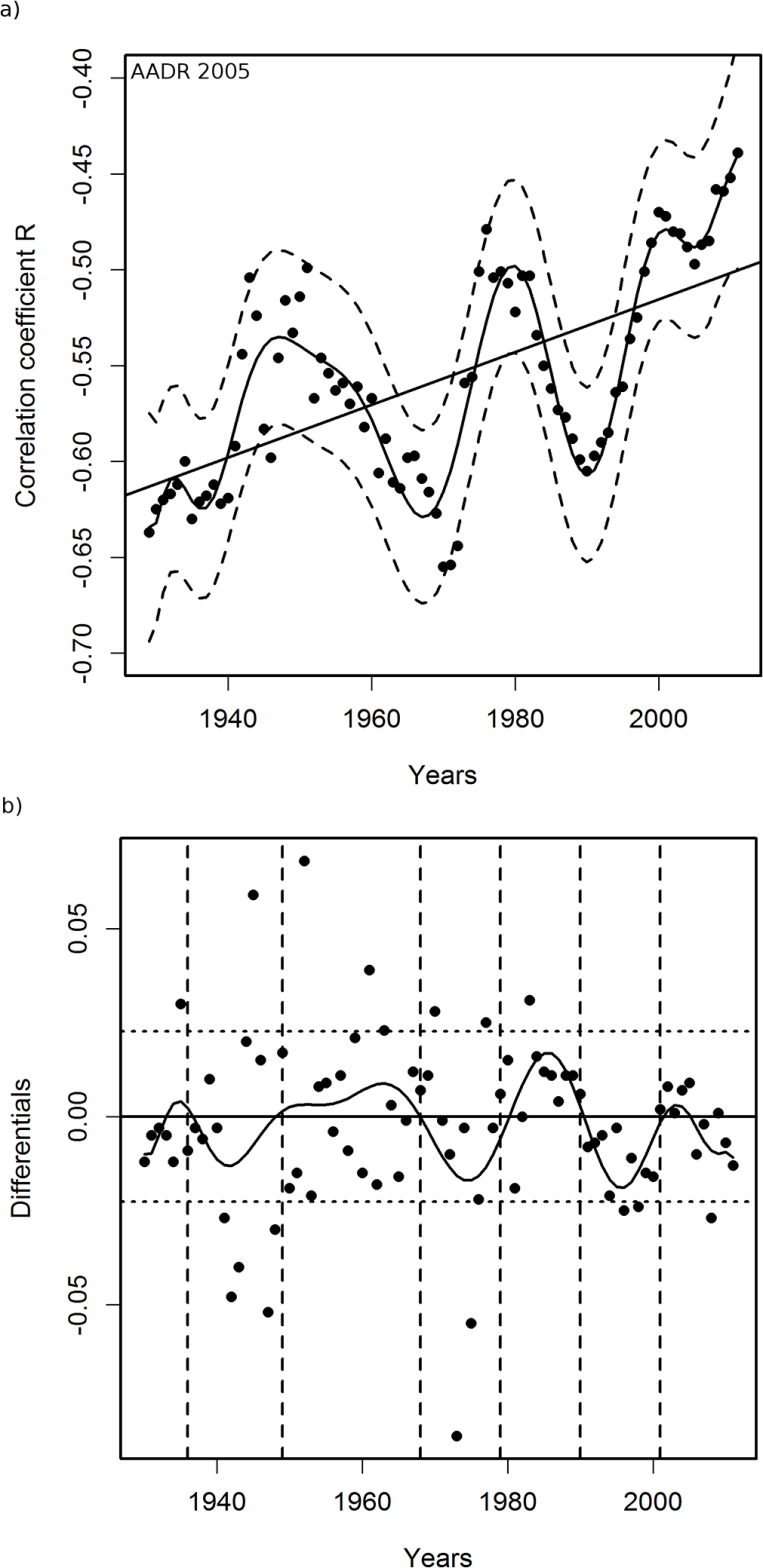
Variability in time of correlation coefficients R for correlations of PCPI (in 49 or 51 states of the USA) with age-adjusted AD death rates (AADRs) for the respective states in 2005. For years 1929–1949 PCPI data were available for 49 states. For years 1950–2011 PCPI data were available for 51 states. Solid line represents the regression curve obtained from polynomial (16^th^ degree) regression with 95% confidence intervals marked by dashed lines.

**Fig 4:**
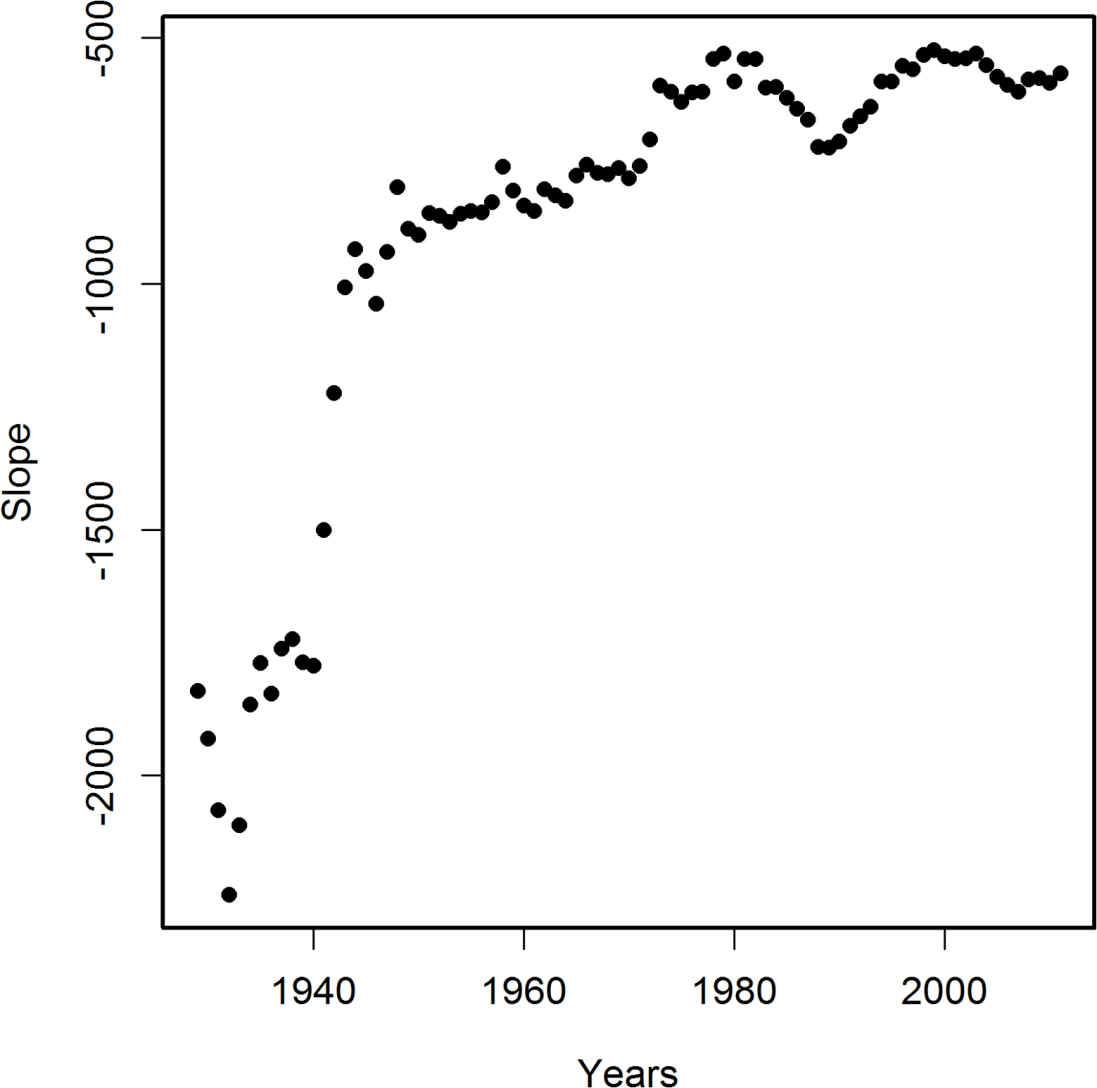
Variability in time of the slope of correlations of PCPI for each state with age-adjusted AD death rates (AADRs) for the respective states in 2005. Numbers of states as in legend to Fig 3.

**Fig 5:**
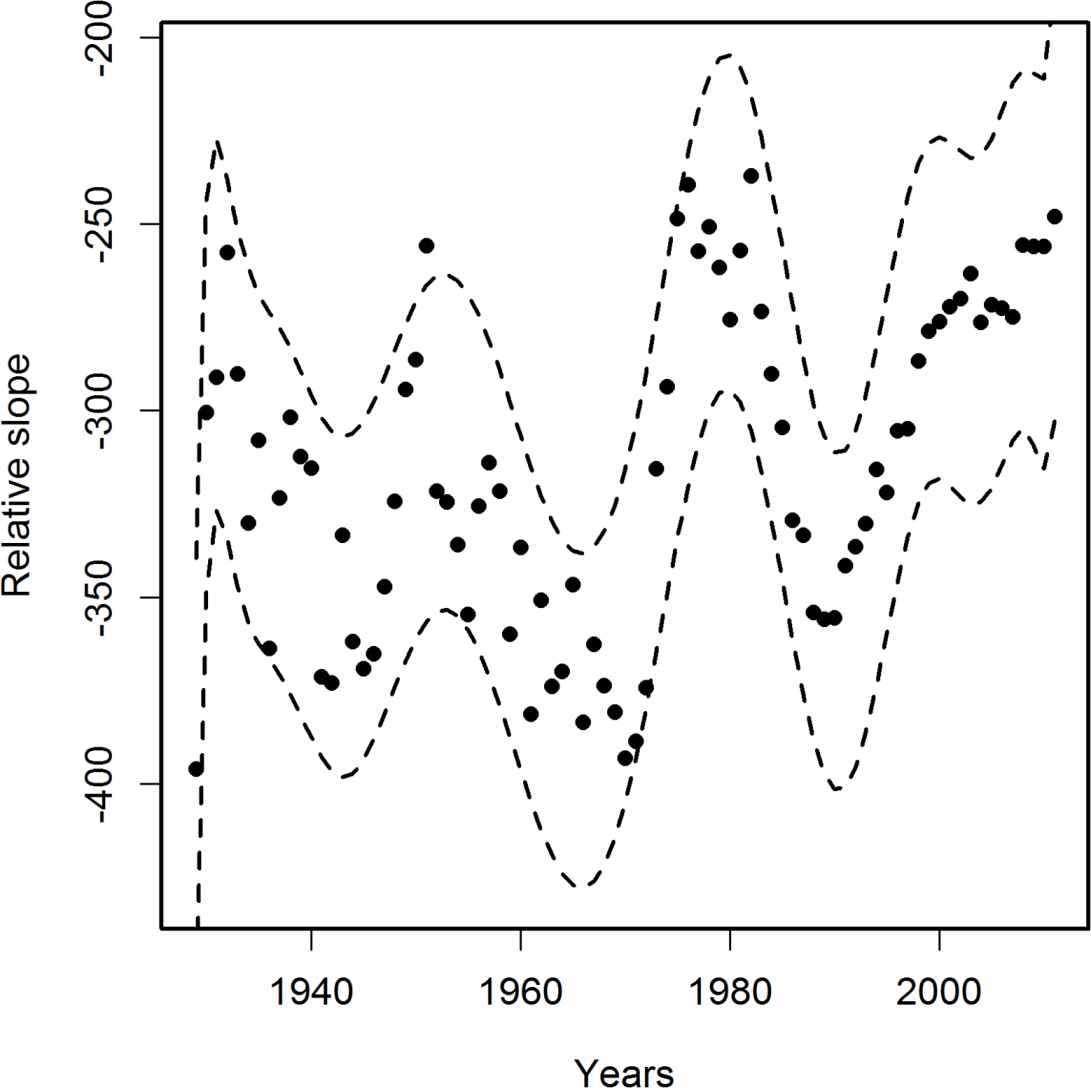
Variability in time of the relative slope of correlations of PCPI for each state with age-adjusted AD death rates (AADRs) for the respective states in 2005. The Relative slope was calculated by dividing slope data from Fig 4 by the coefficient of stratification of PCPI between poorest and richest states; f = PCPI highest/PCPI lowest. This procedure removes the time-dependent influence of stratification of PCPI between states. Solid line regression curve obtained as in Fig 3, dashed lines—95% confidence intervals. The oscillatory character of the relative slope resembles the oscillations of R in Fig 3. Numbers of states as in legend to Fig 3

**Fig 6:**
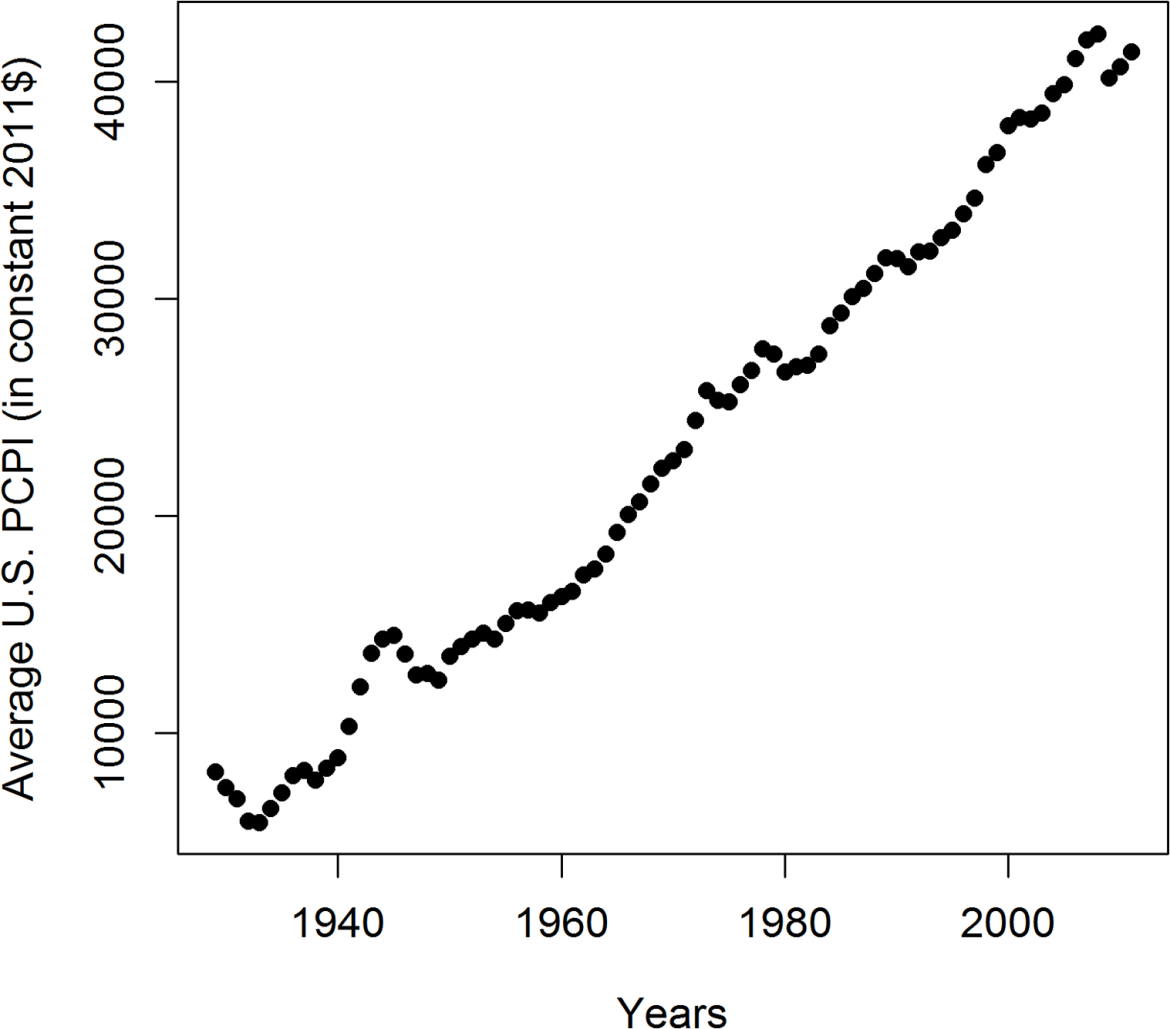
Average US PCPI in years 1929–2011 in constant 2011 dollars. This graph does not explain the variability of R in this period seen in Fig 3.

Fig 1 presents a sample linear regression of PCPI in 1970 by state against corresponding AADRs for 2005. The death rates for other years from the period 2000–2011 correlated with the 1970 PCPIs less strongly than did the 2005 AADRs (Fig 2). Therefore, for further analysis we have chosen AADRs from the year 2005 as those correlating most strongly with PCPIs and, for control calculations, the AADRs from years 2000 and 2008. Table A in S2 File presents the parameters of linear regression of state PCPIs between 1929 and 2011 against AADRs in 2005. Examination of these parameters indicates that the correlations for PCPIs from all years studied are statistically significant. The PCPIs for the years after 2005 were used to check the reliability of calculations. Some residual correlations for years after the patients had died persisted. We interpret this as mostly a result of the year by year similarity of the PCPI data. Data presented in Fig 2 relating PCPIs in 1970 with AADRs in 2000–2008 strengthen this conclusion. The V-shaped curve in Fig 2 reveals a gradual increase in the correlation strength until the maximum in 2005 and a decrease of the strength of the correlation in the years following the maximum. Therefore the income in 1970 influenced the mortality rates both before and after the maximum, which can be interpreted either as a result of the similarity of the income in the consecutive years, a result of age distribution of patients or a chance effect. Since these residual correlations decrease with the time passed since death, we believe that the first explanation is the most likely. Changes of the correlation coefficient R for correlations of PCPI (1929–2011) with AADRs in 2005 plotted against time are shown in Fig 3A The results were regressed by polynomial (16^th^ degree) and 95% confidence intervals calculated from regression results are presented. Additionally, we performed linear regression on these data to visualize the trend line, which appears as a constant decrease of the degree of the correlation with time. The trend line indicates that the level of income at the beginning of the studied period had the highest influence on the AD mortality in 2005. On top of the trend, a remarkable oscillatory character of the regression curve is observed, which is also present in the corresponding data for linear regressions of PCPIs 1929–2011 against AADRs from years 2000 and 2008 (Figs A(a) and B(a) in S2 File). Peaks of the highest and lowest correlations appear for the same years for all three sets of correlations. The 95% confidence intervals allow the conclusion that the observed variability of R is statistically significant. Additionally, differential analysis of results (Figs 3A and A(b),and B(b) in S2 File) confirmed the validity of the observed minima and maxima of R (see statistical methods). This points to the possibility that in the years of extremes of the regression curve, and most probably in the preceding years, the same factors affected the death rates in years 2000, 2005 and 2008. The slope of linear regressions of PCPI 1929–2011 against AADRs in 2005 is plotted in Fig 4. The relation between PCPIs and AADRs (2005) is steepest in the early years of the period analyzed, and the same is true for the 2000 and 2008 AADRs (data not shown). This means that in the early period of life of a potential AD victim a deeper decrease of PCPI was needed to cause one more fatality per 100 000 population in 2005 (as well as in 2000 and 2008), than later in life. We interpret this as the higher ability of a young organism to resist the negative influence of the risk factors related to lower income. On the other hand, the correlation of AADR with income level is stronger in the early periods of life (see Fig 3A, regression curve and trend line), which can be interpreted as a higher importance of the economic status in the early years of life for the late-life health than the status in the midlife and later in life. The observation that the correlation of PCPI with AD death rates changes in time in a statistically significant oscillatory manner prompted us to examine whether this unexpected behavior is a consequence of the oscillations of economic factors or rather is due to long term—presumably biological—consequences mainly related to modifiable risk factors associated with the past income. To answer this question we recalculated the slope data dividing them by a factor Fr = (state PCPI highest in a given year) / (state PCPI lowest in a given year). This procedure cancels out the different year by year stratification of PCPIs between the richest and poorest states. In Fig 5 these corrected slope data (relative slope, regressed as in Fig 3) are plotted against time with corresponding 95% confidence intervals, again giving an oscillatory appearance, which indicates that we are dealing here with true long term biological effects related to the level of past income rather than with chance oscillatory changes of correlations due to economic fluctuations. This oscillatory character of R variability cannot be explained by oscillations of the average US PCPI in constant dollars either (see Fig 6).

## Discussion

Crucial to the evaluation of the quality of the presented results is their potential susceptibility to many possible sources of bias such as, for example, migration of patients between states. Such migration has been estimated to be rather high [31]. However, our results, showing a strong correlation of PCPI with AADRs, suggest only a limited significance of this level of migration. Other possible limitations of using local death rates for analysis of geographical distribution of a disease impact have been discussed by Glymour et al. [31]. Those authors considered them as insignificant. Factors unrelated to personal income such as differences in climate, rural to urban population ratio, stratification of personal income in the population, or average educational attainment or overestimation of death rates due to higher awareness of AD impact on society can also, due to the same reasons as in the case of migration, be considered of limited significance.

The variability of the correlations of PCPI between 1929 and 2005 and the death rates is rather smooth despite the fairly narrow time window of the income data used (one-year state average PCPIs). This indicates that the observed oscillatory character of R changes over time reflects gradual processes which occur in a time span longer than one year. Figs 3–6 indicate that the most plausible interpretation of this phenomenon is a significant influence of lifestyle-related events which occurred in the past rather than temporal economic fluctuations. Among the possible biological factors influencing this correlation, infectious diseases affecting large segments of the population in the past and historical changes in the lifestyle, among them diet, are most likely. Diet, unlike infections, is one of the factors related significantly to the income level and affects statistically the majority of a population. Our studies point to the fact that historical diet changes had a profound influence on the susceptibility of the population to AD. This is not a new observation since the influence of diet on the risk of developing AD is well documented [12,13,14,15]. Our analysis, however, offers some advantages over those earlier studies because, we use whole population data and thus we avoid the problem of selection and recall errors which may bias some of the epidemiological studies.

The mechanism by which life style influences the susceptibility to LOAD most probably relies on epigenetic changes caused by certain diet components and other life style factors [32,33,34]). Since epigenetic changes are reversible, the potential interventional space is likely to be achievable through reversing the undesired epigenetic changes, as has been suggested by some authors [32,34,35,36,37].

A crucial conclusion of our study is that the earliest years of life had the strongest effect on AD mortality in 2005. The years of the Great Depression in the 1930s, when patients who died in 2005 were in their infancy and childhood, markedly shaped their fate as AD victims seven decades later, despite the fact that, as mentioned earlier, at that time of their lives the patients were the most resistant to the negative impact of the risk factors related to low income. The conclusion that early life events shape the late life health status is consistent with numerous observations from studies of the Dutch Hunger Winter consequences and similar studies from other countries (Roseboom et al. [38] and references therein), studies on the influence of the socioeconomic status in childhood on future susceptibility to AD [20,29], and animal studies [39]. In accordance with our analysis are studies by Glymour et al. [31] who observed a relation between the place of birth in the USA and AD mortality. Their observations can be explained by our finding that the relation between PCPI and AD mortality is the strongest for the early life events. The geographic distribution of birthplaces with the highest mortality coincides roughly with the geographic location of low-PCPI states. Since low income is related to poor diet in the early years of life [31] our results are complementary to theirs. Despite the fact that the early-life environmental stimuli have a great impact on late-life health, we observe that also later periods of life contribute to the frequency of death due to AD. This is illustrated by the persisting correlation of the income level with death rates throughout the whole period studied (see Figs 3 and A, and B in S2 File). Interventions reducing the negative influence of poor diet history are therefore possible throughout the whole life of an individual, albeit with a steadily decreasing efficiency. Recently, observations have been published concerning improved cognition of the oldest-old patients due to mental and physical activity training in relation to the non-trained groups [23,24]. These communications are in line with our suggestion that also the oldest patients are responsive to therapy, however, to a lesser extent than younger patients. We postulate that switching to a healthier diet together with mental, social and physical activity interventions can significantly improve the fate of patients. Such interventions are also important for the overall health status of the elderly as reported recently by Rizzuto et al. [40], who performed an 18-year follow up study on 1810 participants from the Kungsholmen district of Stockholm and found that a low-risk lifestyle profile can add five years to a woman’s and six years to a man’s life. A similar but lesser effect was also observed for the 85 and older group.

In the light of our results, the differences in AD mortality rates between races mentioned in the first paragraph of Rationale can be largely explained by differences in per capita income, since populations of African-Americans and Hispanic-origin Americans have lower per capita incomes than whites (US Census Bureau 2010 ACS 1 Year Estimates). Race-specific diets and other lifestyle factors, e.g., educational attainment (US Census Bureau 2010 ACS 1 Year Estimates) or others, related to the income may contribute to racial differences in AD mortality rates.

We have shown that the novel whole-population approach used here gives valuable results allowing identification of the long-gone causes of the enormous increase in the prevalence of AD in the USA in recent years. This was possible owing to the use of a general parameter of the healthiness of the lifestyle of a population; in our case the historical data of PCPI, and their correlation with death rates. Changing the habits of a population toward more healthy nutrition and other beneficial lifestyle behaviors, such as physical, mental and social activity, appear as the most effective measures to be undertaken to combat this disease. A similar strategy of reducing AD prevalence in the population by reducing the influence of certain risk factors has been proposed by Barnes and Yaffe [41]. A recently published paper by Grant [42] uses a similar logic to ours of analyzing the effects of historical changes of diet on the present AD mortality in Japan and eight developing countries. The author concludes, in line with our results, that the dietary history of these countries has affected the present AD prevalence. Our approach of relating semi-quantitatively the history of a population’s lifestyle with the present mortality data could be easily applied to diseases other than AD, such as cancer, diabetes, cardiovascular disease and others in the hope of finding more efficient strategies of prevention.

## Supporting Information

S1 File Dataset with Excel spreadsheet containing PCPI and normalized PCPI data (XLS)

S2 File Fig A in S2 File Variability in time of correlation coefficients R for correlations between US state PCPIs in the period 1929–2005 and age-adjusted AD death rates (AADRs) for the respective states in 2000. Fig B in S2 File Variability in time of correlation coefficients R for correlations of US states’ PCPIs in period 1929–2005 against age-adjusted AD death rates (AADRs) for these states in 2008. Table A in S2 File Parameters of correlation between PCPIs for each state of the USA and AADRs in 2005. Table B. The Kendall’s Tau correlation coefficients and results of the Mann-Kendall trend test for the correlations between PCPI and age-adjusted AD death rates (AADRs) in 2005 (p value)
(DOC)
