# Supplementary material for: Correlation of Alzheimer’s Disease Death Rates with Historical Per Capita Personal Income in the USA
Source: PLoS One. 2015 May 11;10(5):e0126139. doi: 10.1371/journal.pone.0126139 (PMC4427436; doi:10.1371/journal.pone.0126139)
Supplement: S2 File — Fig B in S2 File Variability in time of correlation coefficients R for correlations of US states’ PCPIs in period 1929–2005 against age-adjusted AD death rates (AADRs) for these states in 2008. Table A in S2 File Parameters of correlation between PCPIs for each state of the USA and AADRs in 2005. Table B. The Kendall’s Tau correlation coefficients and results of the Mann-Kendall trend test for the correlations between PCPI and age-adjusted AD death rates (AADRs) in 2005 (p value) (DOC) [file pone.0126139.s002.doc]

**Correlation of Alzheimer’s disease death rates with historical per capita personal income in the USA.**

**Supporting Information**

Dariusz Stępkowski^1^, Grażyna Woźniak^1^, Marcin Studnicki^2^

^1^Laboratory of Molecular Basis of Cell Motility, Nencki Institute of Experimental Biology, Warszawa, Poland

^2^Department of Experimental Design and Bioinformatics, Warsaw University of Life Sciences-SGGW, Warszawa, Poland

List of content:

1. Legends to Supplementary Figures
2. Fig. A
3. Fig. B
4. Table A
5. Table B

**Legends to the figures**

Fig. A. **Variability in time of correlation coefficients R for correlations between US state PCPIs in the period 1929-2005 and age-adjusted AD death rates (AADRs) for the respective states in 2000**. Numbers of states as in legend to Fig. 3. a) Solid line represents polynomial (16^th^ degree) regression curve, dashed lines represent 95% confidence intervals. b) differential analysis of results presented in a). Regression line crossing zero indicate minima and maxima of regression line in a).

Fig. B. **Variability in time of correlation coefficients R for correlations of US states’ PCPIs in period 1929-2005 against age-adjusted AD death rates (AADRs) for these states in 2008**. Numbers of states as in legend to Fig. 3. a) Solid line represents polynomial (16^th^ degree) regression curve, dashed lines represent 95% confidence intervals. b) differential analysis of results presented in a). Points where the regression line crosses zero indicate minima and maxima of the regression line in a).

Fig. A


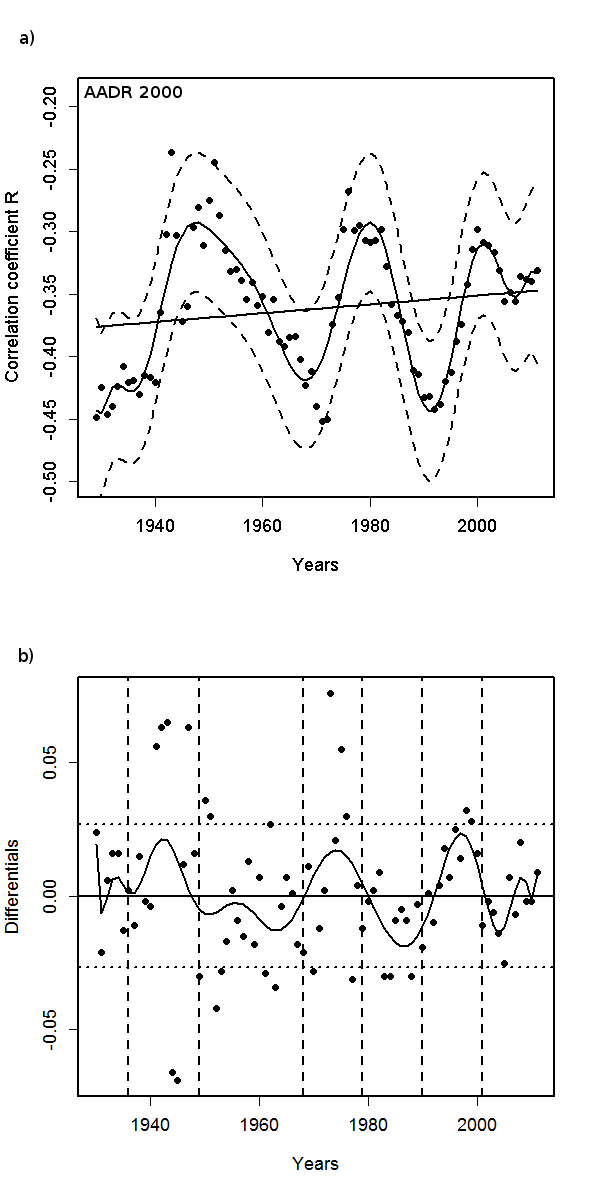


Fig. B


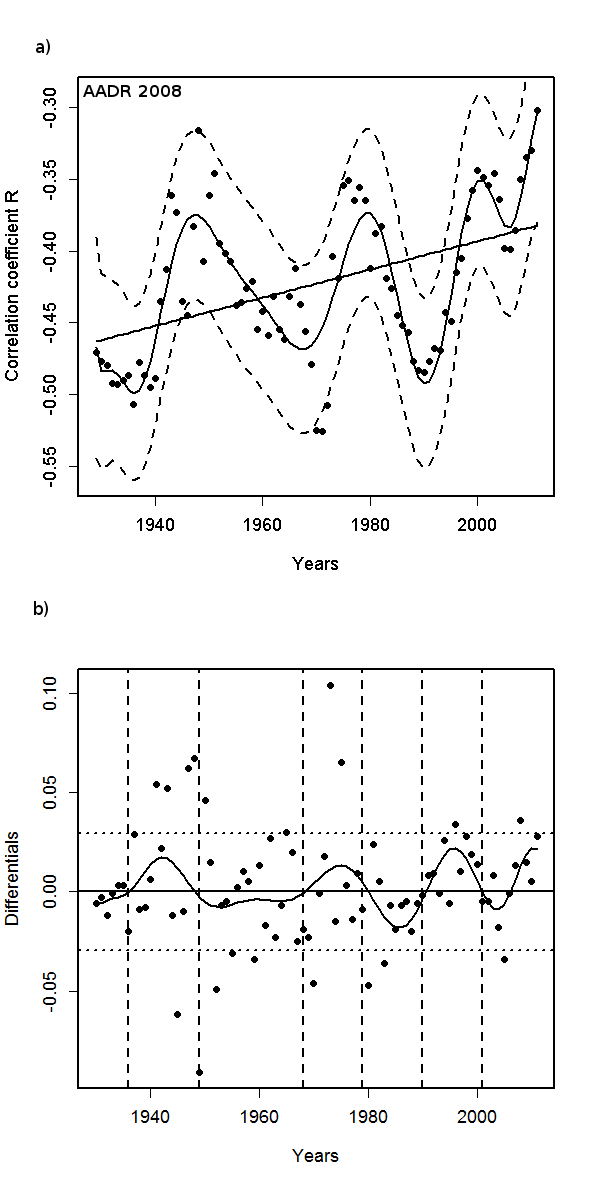


Table A. **Parameters of correlation between PCPIs for each state of the USA and AADRs in 2005.** PCPI for each state in 1929-2005 was normalized by multiplication by the average US PCPI for a given year and dividing by the average US PCPI in 2011. Linear regression was performed using Origin 7.5 software. Analysis for 49 states for years 1929-1949 (without Alaska and Hawaii) and for 51 states for years 1950-2011. R – correlation coefficient, p – probability (that R is zero), slope of the regression line, SD – standard deviation of slope determination.

| **Years** | **R** | **p** | **slope** | | **±**SD |
| --- | --- | --- | --- | --- | --- |
| | 1929 | | --- | | 1930 | | 1931 | | 1932 | | 1933 | | 1934 | | 1935 | | 1936 | | 1937 | | 1938 | | 1939 | | 1940 | | 1941 | | 1942 | | 1943 | | 1944 | | 1945 | | 1946 | | 1947 | | 1948 | | 1949 | | 1950 | | 1951 | | 1952 | | 1953 | | 1954 | | 1955 | | 1956 | | 1957 | | 1958 | | 1959 | | 1960 | | 1961 | | 1962 | | 1963 | | 1964 | | 1965 | | 1966 | | 1967 | | 1968 | | 1969 | | 1970 | | 1971 | | 1972 | | 1973 | | 1974 | | 1975 | | 1976 | | 1977 | | 1978 | | 1979 | | 1980 | | 1981 | | 1982 | | 1983 | | 1984 | | 1985 | | 1986 | | 1987 | | 1988 | | 1989 | | 1990 | | 1991 | | 1992 | | 1993 | | 1994 | | 1995 | | 1996 | | 1997 | | 1998 | | 1999 | | 2000 | | 2001 | | 2002 | | 2003 | | 2004 | | 2005 | | 2006 | | 2007 | | 2008 | | 2009 | | 2010 | | 2011 | | | -0.637 | | --- | | -0.625 | | -0.62 | | -0.617 | | -0.612 | | -0.6 | | -0.63 | | -0.621 | | -0.618 | | -0.612 | | -0.622 | | -0.619 | | -0.592 | | -0.544 | | -0.504 | | -0.524 | | -0.583 | | -0.598 | | -0.546 | | -0.516 | | -0.533 | | -0.514 | | -0.499 | | -0.567 | | -0.546 | | -0.554 | | -0.563 | | -0.559 | | -0.57 | | -0.561 | | -0.582 | | -0.567 | | -0.606 | | -0.588 | | -0.611 | | -0.614 | | -0.598 | | -0.597 | | -0.609 | | -0.616 | | -0.627 | | -0.655 | | -0.654 | | -0.644 | | -0.559 | | -0.556 | | -0.501 | | -0.479 | | -0.504 | | -0.501 | | -0.507 | | -0.522 | | -0.503 | | -0.503 | | -0.534 | | -0.55 | | -0.562 | | -0.573 | | -0.577 | | -0.588 | | -0.599 | | -0.605 | | -0.597 | | -0.59 | | -0.585 | | -0.564 | | -0.561 | | -0.536 | | -0.525 | | -0.501 | | -0.486 | | -0.47 | | -0.472 | | -0.48 | | -0.481 | | -0.488 | | -0.497 | | -0.487 | | -0.485 | | -0.458 | | -0.459 | | -0.452 | | -0.439 | | | 0.0001 | | --- | | 0.0001 | | 0.0001 | | 0.0001 | | 0.0001 | | <0.0001 | | 0.0001 | | 0.0001 | | 0.0001 | | 0.0001 | | <0.0001 | | <0.0001 | | <0.0001 | | <0.0001 | | 0.0002 | | 0.0001 | | <0.0001 | | 0.0001 | | <0.0001 | | 0.0001 | | <0.0001 | | 0.0001 | | 0.0002 | | <0.0001 | | <0.0001 | | <0.0001 | | <0.0001 | | <0.0001 | | <0.0001 | | <0.0001 | | <0.0001 | | <0.0001 | | <0.0001 | | <0.0001 | | <0.0001 | | <0.0001 | | <0.0001 | | <0.0001 | | <0.0001 | | <0.0001 | | <0.0001 | | 0.0001 | | <0.0001 | | <0.0001 | | <0.0001 | | <0.0001 | | 0.0002 | | 0.0004 | | 0.0002 | | 0.0002 | | 0.0001 | | <0.0001 | | 0.0004 | | 0.0002 | | <0.0001 | | <0.0001 | | <0.0001 | | <0.0001 | | <0.0001 | | <0.0001 | | <0.0001 | | <0.0001 | | <0.0001 | | <0.0001 | | <0.0001 | | <0.0001 | | <0.0001 | | <0.0001 | | <0.0001 | | -0.0002 | | 0.0003 | | 0.0005 | | 0.0005 | | 0.0004 | | 0.0004 | | 0.0003 | | 0.0002 | | 0.0003 | | 0.0003 | | 0.0007 | | 0.0007 | | 0.0009 | | 0.0128 | | | -1827 | | --- | | -1924 | | -2070 | | -2242 | | -2100 | | -1855 | | -1771 | | -1833 | | -1742 | | -1722 | | -1769 | | -1777 | | -1501 | | -1222 | | -1007 | | -930 | | -974 | | -1040 | | -935 | | -803 | | -888 | | -900 | | -856 | | -861 | | -874 | | -857 | | -852 | | -854 | | -834 | | -761 | | -810 | | -840 | | -852 | | -807 | | -820 | | -831 | | -780 | | -758 | | -774 | | -777 | | -764 | | -785 | | -760 | | -706 | | -597 | | -609 | | -630 | | -611 | | -609 | | -543 | | -532 | | -589 | | -542 | | -542 | | -601 | | -600 | | -621 | | -644 | | -666 | | -721 | | -723 | | -710 | | -679 | | -659 | | -640 | | -589 | | -588 | | -557 | | -563 | | -535 | | -525 | | -537 | | -542 | | -541 | | -532 | | -555 | | -579 | | -595 | | -609 | | -584 | | -581 | | -591 | | -572 | | | ±322 | | --- | | ±351 | | ±382 | | ±418 | | ±395 | | ±361 | | ±316 | | ±338 | | ±323 | | ±325 | | ±325 | | ±329 | | ±298 | | ±275 | | ±252 | | ±221 | | ±198 | | ±203 | | ±209 | | ±194 | | ±206 | | ±214 | | ±212 | | ±199 | | ±192 | | ±184 | | ±179 | | ±181 | | ±172 | | ±161 | | ±161 | | ±165 | | ±160 | | ±158 | | ±152 | | ±153 | | ±149 | | ±146 | | ±144 | | ±142 | | ±136 | | ±129 | | ±126 | | ±120 | | ±126 | | ±130 | | ±155 | | ±160 | | ±149 | | ±134 | | ±129 | | ±137 | | ±133 | | ±133 | | ±136 | | ±130 | | ±131 | | ±131 | | ±135 | | ±142 | | ±138 | | ±134 | | ±131 | | ±129 | | ±127 | | ±123 | | ±124 | | ±125 | | ±130 | | ±132 | | ±135 | | ±144 | | ±145 | | ±141 | | ±139 | | ±142 | | ±144 | | ±153 | | ±157 | | ±162 | | ±161 | | ±167 | | ±167 | | |

Table B. **The Kendall’s Tau correlation coefficients and results of the Mann-Kendall trend test for the correlations between PCPI and age-adjusted AD death rates (AADRs) in 2005 (p value )**

| Segments (seasons) | Kendall’s Tau correlation | | Mann-Kendall test | |
| --- | --- | --- | --- | --- |
|  | τ | p value | S | p value |
| 1929-2011 (all years) | 0.423 | <0.001 | 5.666 | <0.001 |
| 1929 -1936 | 0.357 | 0.216 | 1.237 | 0.216 |
| 1937-1950 | 0.538 | 0.007 | 2.682 | 0.007 |
| 1951-1969 | -0.762 | <0.001 | -4.561 | <0.001 |
| 1970-1980 | 0.55 | 0.018 | 2.357 | 0.018 |
| 1981-1991 | -0.917 | <0.001 | -3.928 | <0.001 |
| 1992-2002 | 0.891 | <0.001 | 3.815 | <0.001 |
| 2003-2011 | 0.667 | 0.012 | 2.502 | 0.012 |

The Mann-Kendall test shows that there are significant positive trends for correlations between PCPI and AADR across all years. Only for the first period (1929-1936) no significant monotonic trend was observed. Segments 1951-1969 and 1981-1991 show negative significant trends. The negative trend was strongest between 1981 and 1991 (τ = -0.917). Other segments were characterized by significant positive trends. For the seasons 1937-1950 and 1970-1980 the correlations between PCI and AADR exhibit the weakest trends (τ = 0.538 and τ = 0.550, respectively).
